# Reduced Ectopic Pregnancy Rate on Day 5 Embryo Transfer Compared with Day 3: A Meta-Analysis

**DOI:** 10.1371/journal.pone.0169837

**Published:** 2017-01-25

**Authors:** Bingqian Zhang, Linlin Cui, Rong Tang, Lingling Ding, Lei Yan, Zi-Jiang Chen

**Affiliations:** 1 Center for Reproductive Medicine, Shandong Provincial Hospital Affiliated to Shandong University, Jinan, China; 2 National Research Center for Assisted Reproductive Technology and Reproductive Genetics, Jinan, China; 3 The Key laboratory of Reproductive Endocrinology (Shandong University), Ministry of Education, Jinan, China; 4 Center for Reproductive Medicine, Ren Ji Hospital, School of Medicine, Shanghai Jiao Tong University, Shanghai, China; 5 Shanghai Key Laboratory for Assisted Reproduction and Reproductive Genetics, Shanghai, China; Institute of Zoology Chinese Academy of Sciences, CHINA

## Abstract

**Objective:**

To compare the risk of ectopic pregnancy (EP) after embryo transfer on day 3(D3-ET) and day 5(D5-ET).

**Design:**

Meta-analysis

**Patients:**

Women with pregnancy resulting from in vitro undergoing in vitro fertilization (IVF) or intracytoplasmic sperm injection (ICSI)

**Result(s):**

Twenty-two studies were identified through research conducted using the PubMed, Embase, and Cochrane databases and ClinicalTrials.gov. All studies were conducted prior to October 2016. Adding the reproductive data from our center, a total of 143 643 pregnancies were reviewed(D3-ET: n = 62027,D5-ET:n = 81616). A lower EP rate was found in women undergoing D5-ET than in those undergoing D3-ET [relative risk (RR), 0.67;95% confidence interval (CI), 0.54–0.85;143643 pregnancies in 23 studies; I^2^ = 67%]. These results were validated in subgroups of fresh embryo-transfer (Fre-ET) cycles [RR, 0.78; 95%CI, 0.69–0.88; 91 871 pregnancies in 21 studies; I^2^ = 29%] and frozen-thawed embryo-transfer (Fro-ET) cycles [RR, 0.43; 95%CI, 0.36–0.51; 51 772 pregnancies in 10 studies; I^2^ = 33%]. After separating out the randomized controlled trials (RCTs), a significant difference was found in the retrospective studies in both subgroups [both Fre-ET (RR,0.78;95% CI 0.69–0.88);91182 pregnancies in 14 studies; I^2^ = 45%] and Fro-ET(RR,0.43;95% CI 0.36–0.51; 51751pregnancies in 9 studies;I^2^ = 33%)], while the RCTs showed no statistical significance for Fre-ET cycles[RR,0.86;95% CI 0.32–2.26); 689 pregnancies in 7 studies; I^2^ = 0%].

**Conclusion(s):**

The present study indicates that D5-ET reduces the risk for EP in cycles that use IVF or ICSI, compared with D3-ET. It suggests that D5-ET may be a better choice for decreasing the EP rate in assisted reproductive technology. Further high-quality randomized controlled trials are anticipated.

## Introduction

Ectopic pregnancy (EP) is a life-threatening clinical gynecologic emergency [1]. Hypovolemic shock resulting from EP rupture is the primary cause of death during early pregnancy [2]. The rate of EP in assisted reproductive technology (ART) reportedly ranges from 1.6% to 8.6%, 4 times higher than with natural conception [3–6].

In nature, the fertilized ovum undergoes cleavage as it passes down the Fallopian tube. The ovum enters the uterine cavity about 3 to 4 days after fertilization (day 3–4) and then forms a single, large cavity as fluid enters and occupies the intercellular spaces; the resulting structure is called a blastocyst. On about day 6 or 7, the blastocyst penetrates the epithelial cells of the uterine mucosa [7]. During in vitro fertilization(IVF), the transferred day 3 (D3-ET) embryos will not implant immediately, and may be transported back into the Fallopian tube via the retrograde contractions of the uterine muscular layer, leading to ectopic implantation [7].Therefore, performing embryo transfer on day 5 (D5-ET) can shorten the “wandering” time of the embryo and therefore hypothetically reduce the risk for EP compared with traditional ET on day 3.

Previous studies indicate that the EP rate in blastocyst-transfer cycles is significantly lower than in cleavage-transfer cycles during IVF or intracytoplasmic sperm injection (ICSI)[8–11]. However, the conclusions of these studies are not consistent. Several studies do not find a statistically significant difference in the EP rate between D3-ET and D5-ET [12, 13]. In fact, the opposite results are described by Keegan and Rosman [14, 15], who show that the EP rate is increased in D5-ET compared with D3-ET.

The aim of this study is to elucidate, using a meta-analysis structure, whether the risk of EP may be reduced using D5-ET.

## Method and Materials

### Literature Search

Two authors (BQ Z and LL C) independently performed the literature search in the online databases (PUBMED, EMBASE, COCHRANE and Clinical Trials. gov) up to October 2016. A search strategy was carried out based on key words and medical subject heading (MeSH) terminology: embryo transfer, IVF, day 3(or three), day 5(or five), cleavage stage, blastocyst stage, ectopic pregnancy and heterotopic pregnancy. We also hand searched the reference listed in the related reviews and articles.

### Outcome Measures

EP was defined as a pregnancy with an extra uterine gestational sac, or as the absence of an intrauterine gestational sac but increasing human chorionic gonadotropin (hCG) levels[16]. Heterotopic pregnancy, diagnosed as EP co-existing with a synchronous clinical intrauterine pregnancy, was also grouped into EP in this analysis. The EP rate was calculated as per number of clinical pregnancies.

### Study Selection

Two authors (BQ Z and LL C) independently assessed data selection. Where there were any queries, a third author (L Yan) was consulted to discuss any disagreements. The criteria for included studies were: (i) English papers;(ii) compare the EP rate between D3-ET and D5-ET groups. PGD/PGS cycles were excluded. We simply intake the latest and the largest dataset when the studies were overlapped.

### Data Extraction and Quality Assessment

We generated characteristic and results forms for the included studies (included the retrospective data in our institute during 2010–2015). The data were extracted by two review authors (BQ Z and LL C) independently according to the selection criteria. Any disagreements were resolved by discussion with another review author (Yan L). We extracted statistical data from the original papers. Seventeen authors were contacted by e-mail to request further data regarding EP rate, with responses received for 2 unpublished datasets.

Furthermore, retrospective data of our institute (Center for Reproductive Medicine, Shandong University) during 2010–2015 were complemented for the meta-analysis. A total of 31115 clinical pregnancies aged from 19 to 49 were recruited. All of the pregnancies included were from IVF or ICSI cycles. Among them, 20347 pregnancies were of D3-ET and 10768 of D5-ET. The total number of EP is 654. Written informed consents were obtained from all the participants. The recruitment of our data was approved approved by the institutional review board of Center for Reproductive Medicine of Shandong University. Characteristics of our data were listed in S1 Table.

Information regarding authorship, publication dates, journals, sample size, location and duration was recorded. All the accessible data were extracted into REVMAN 5.3 for further analysis.

The risk of bias of the included randomized controlled trials (RCTs) were evaluated according to “*Cochrane Handbook for Systematic Reviews of Interventions*[17]”. Six related domains were assessed in each included trial:1) random sequence generation;2) allocation concealment;3) blinding of participants and personnel;4) incomplete outcome data; 5) selective reporting;6) other bias. Each item was judged as a rating of “low risk”, “unclear risk” and “high risk” of bias. The evaluation results were shown in S1 Fig. The retrospective cohort studies and case-control studies were evaluated according to “*the Newcastle–Ottawa Scale(NOS)*[18]*”*, which were rated based on 8 items and categorized in 3 domains: study subject selection (4 items), comparability between groups (1 items) and outcome measure (3 items). Scores were represented with stars for each quality item and the total score of this assessment is nine. (S2 and S3 Tables). Study design (RCTs, retrospective cohort or case control study) were evaluated as part of assessment of the risk of bias across the studies.

### Statistics

Statistical heterogeneity was evaluated by the measure of the I^2^. We used a fixed-effects model in the cases that the I^2^ ≤ 50%, which indicate a low or moderate heterogeneity. Otherwise, a random-effects analysis model was developed instead. Subgroup analysis was conducted according to the confounding factor of embryo frozen.

Sensitivity analysis were applied in 3 methods to determine the stability of outcome: repeating meta-analysis with RCTs with low risk of bias by allocation concealment and a NOS score at nine; omitting studies that had day2/3 or day 5/6 embryo transfer; omitting a single study in turn. Sensitivity analysis was performed using Stata 12.0.

Potential publication bias were assessed by Begg's funnel plots and Egger's linear regression tests[19].Begger’s funnel plots and Egger's linear regression test were performed using Stata 12.0. The number of events was pooled into the RevMan5.3 for Mac and analyzed using the modified Mantel-Haenzel method. The summary measures were reported as relative risk(RR) with 95% confidence interval (CI). P <0.05 was considered statistically significant.

The meta-analysis was reported following the Preferred Reporting Item for Systematic Reviews and Meta-analyses (PRISMA) statement[20](S1 File).

## Results

### Literature Search

**Fig 1:**
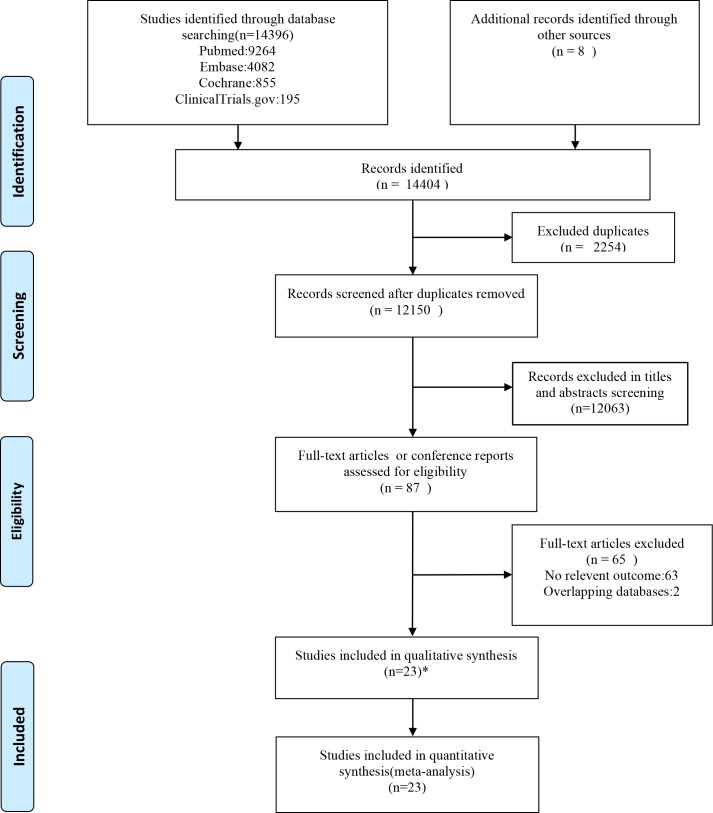
Summary of study selection. Summary of study selection. * included the retrospective study in our institute

A total of 12150 articles were identified. After exclusion Forest plot of EP rate for embryo transfer on day 3 versus day 5 based on titles and abstracts, 87 published papers of full texts and conference reports were screened (Fig 1). We excluded 65 studies for no relevant outcome and overlapped data. Finally, a total of 143643 pregnancies and 2734 events (diagnose of EP) from 23 studies (22 literature researches and retrospective data of our institute) were included in the meta-analysis.

### Study Characteristics

**Fig 2:**
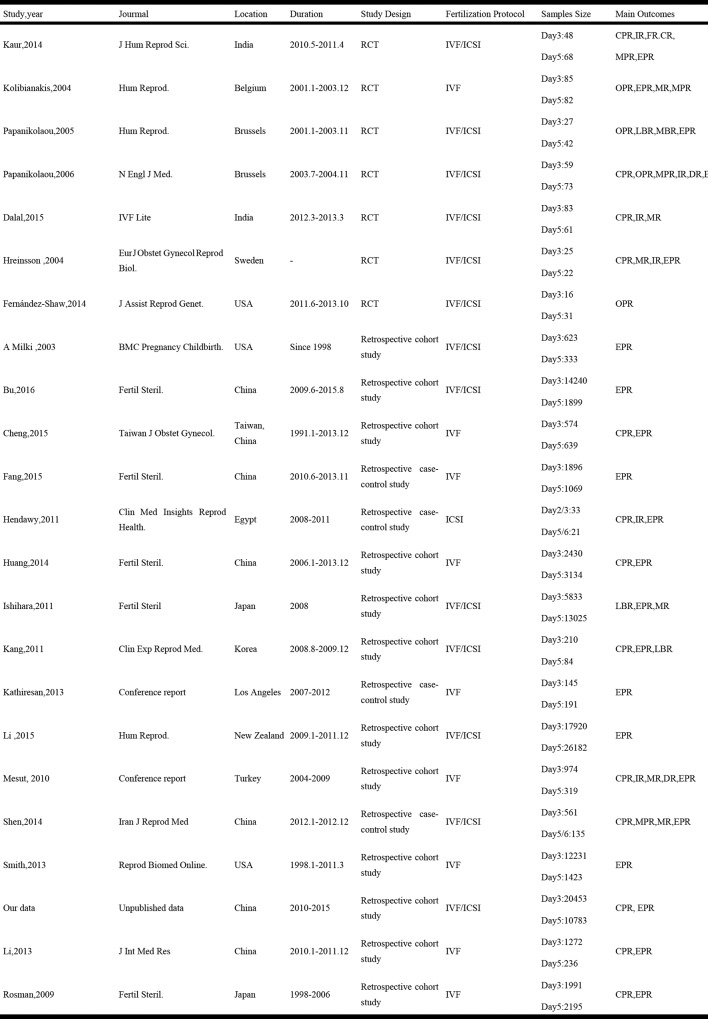
Characteristics of all studies included in the systematic review. RCT: randomized controlled trial; IVF: in vitro fertilization; ICSI: intracytoplasmic sperm injection; CPR: Clinical pregnancy rate; EPR: ectopic pregnancy rate; OPR: Ongoing pregnancy rage; LBR: Live birth rate; MR: Miscarriage rate; DR: Delivery rate; IR: Implantation rate.

Characters of the 23 eligible studies were listed in Fig 2, of which two were conference reports [21, 22], seven were RCTs[23–29], and sixteen were retrospective studies[twelve cohort [8, 10–14, 21, 30–33] and 4 case-control studies[9, 22, 34, 35]] (Fig 2). Thirty-three studies were conducted in combined IVF/ICSI cycles[8, 11, 13, 23–28, 31, 33, 34],9 studies were in IVF [9, 10, 12, 14, 21, 22, 29, 30, 32]and one study were only in ICSI cycles[35]. All studies compared the EP rate between day 3 versus day 5 except two[34, 35], which compared day2/3 versus day 5/6.Embryos transferred on day2/3 were grouped to D3-ET group and embryos transferred on day5/6 were grouped to D5-ET group. All studies except two [9, 13]stated the number of embryo transfer in their studies. A fixed number of single embryo transfer (SET)was in 4 studies[8, 24, 28, 31] and a number of two (DET)was in 1 study[25]. A maximum of three embryos were transferred in both groups in the remaining 16 studies, from which a statistical significance was found in one study[21]. The dataset of donor egg cycles was provided in one study[12](Fig 2).

Systematic risk assessment of methodological bias of included RCTs revealed three RCTs did not[23,26,27]clearly describe an acceptable method of sequence generation and five RCTs[23,25–28]did not clearly describe their methods of allocation concealment, therefore, we rated them at unclear risk of bias. We rated one study [28] at unclear risk in attribution bias domain, because the general random patients were not reported and we were unable to determine the integrity of data. We assessed three studies [24,26,28]at an unknown risk of other bias, for an insufficient information about basic characters (S1 Fig).By assessment using the NOS of retrospective studies, 6 studies [9, 12, 30, 32, 33, 36]were awarded nine scores, while the others were awarded with eight scores (S2 and S3 Tables)

### Ectopic Pregnancy

**Fig 3:**
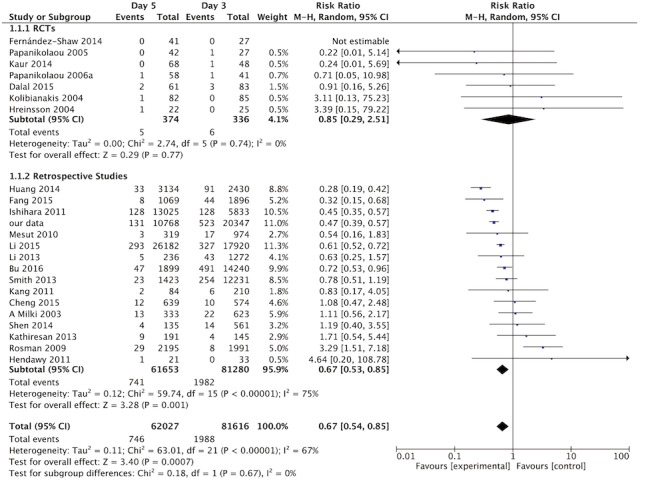
Forest plot of EP rate for embryo transfer on day 3 versus day 5. Forest plot of EP rate for embryo transfer on day 3 versus day 5 in general, as well as in RCTs and reproductive studies separately.

A total of 143643 pregnancies were reviewed (D3-ET: n = 62027, D5-ET: n = 81616). The sample size of each study ranged from 47 to 44102. There was a significant decreased risk of EP on D5-ET than D3-ET in total pregnancies [RR,0.67; 95% CI, 0.54–0.85;143643 pregnancies in 23 studies; I^2^ = 67%](Fig 3). After RCTs separated, significant difference was still found in observation studies[RR,0.67; 95% CI,0.53–0.85;142933 pregnancies 16 studies; I^2^ = 75%]. Although a general trend of lower EP risk on D5-ET was showed, it did not reach a statistical significance in RCTs[RR,0.85; 95% CI, 0.29–2.51;710 pregnancies in 7 studies; I^2^ = 0%](Fig 3).

**Fig 4:**
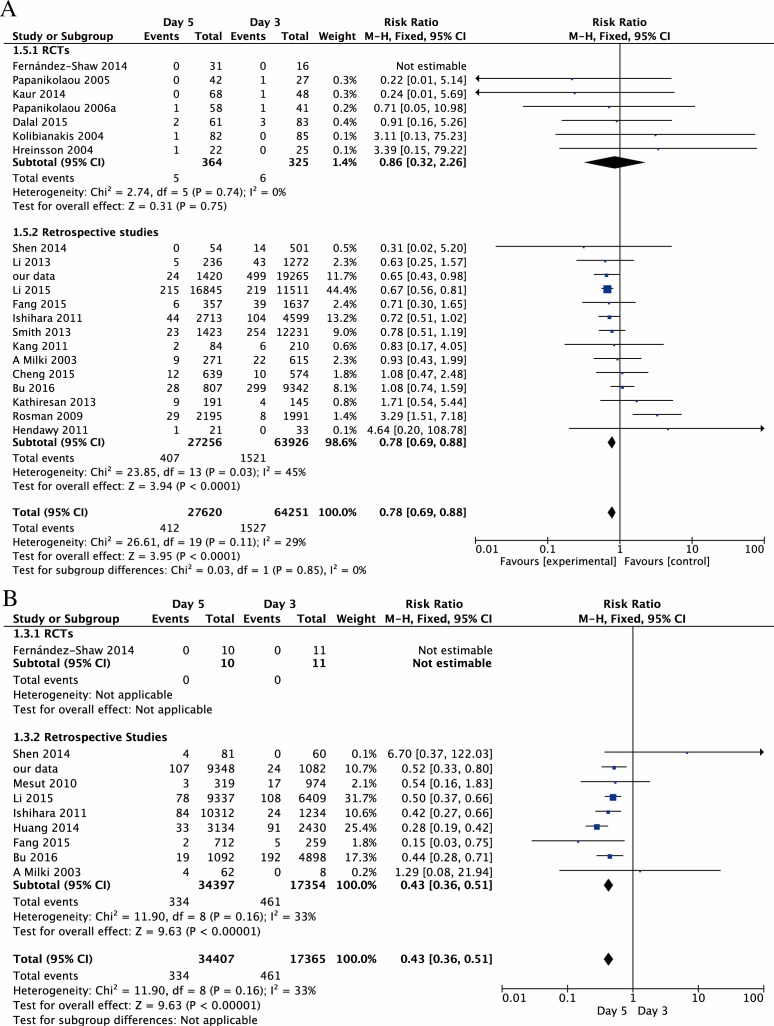
Forest plot of EP rate for embryo transfer on day 3 versus day 5 in subgroups. Forest plot of EP rate for embryo transfer on day 3 versus day 5 in subgroups. **A.** Forest plot of EP rate for embryo transfer in fresh cycles on day 3 versus day 5 in general, as well as in RCTs and reproductive studies separately. **B.** Forest plot of EP rate for embryo transfer in frozen-thawed cycles on day 3 versus day 5 in general, as well as in RCTs and reproductive studies separately.

As the above results provided a high heterogeneity (I^2^>50%),we pooled the datasets from the original full text and classified them into the fresh embryo transfer (Fre-ET) and frozen-thawed embryo transfer (Fro-ET) cycles de novo. D5-ET showed lower EP rate compared to D3-ET in both Fre-ET cycles [RR,0.78;95% CI, 0.69–0.88; 91871 pregnancies in 21 studies; I^2^ = 29%] and Fro-ET cycles [RR,0.43;95% CI,0.36–0.51; 51772 pregnancies in 10 studies, I^2^ = 33%] (Fig 4). After RCTs separated, significantly difference was still found in retrospective studies in both subgroups of Fre-ET cycles[RR,0.78;95% CI, 0.69–0.88; 91182 pregnancies in 14 studies; I^2^ = 45%] and Fro-ET cycles[RR,0.43;95% CI, 0.36–0.51; 51751 pregnancies in 9 studies;I^2^ = 33%], while no statistical significance was found in RCTs of Fre-ET cycles only[RR,0.86;95% CI, 0.32–2.26; 689 pregnancies in 7 studies; I^2^ = 0%](Fig 4).

### Sensitivity Analysis

Sensitivity analysis with 8 studies of low bias risk by allocation concealment and a NOS score at nine did not substantially influence our findings [RR,0.65; 95% CI 0.56–0.76,79885 pregnancies in 8 studies; I^2^ = 10%][9, 12, 24, 29, 30, 32, 33, 36](data not shown). Sensitivity analysis excluding studies of day2/3 or day 5/6 ET [34, 35] obtained similar results [RR, 0.65; 95% CI,0.52–0.82; 142893 pregnancies in 21 studies, I^2^ = 68%](data not shown). Omitting single study in turn did not significantly alter the initial association of EP rate between D3-ET and D5-ET(S2 Fig).

### Publication Bias

Visual inspection of Begg’s funnel plots did not suggest obvious publication bias on study findings Publication Bias. The Egger's linear regression test also indicated no evidence of publication bias among studies of ART and adverse obstetric outcomes (P = 0.10 for total EP)(S3 Fig).

## Discussion

The current meta-analysis suggests that the EP rate is significantly lower with D5-ET than with D3-ET. Similar results are found in both the Fre-ET and Fro-ET subgroups.

One explanation may relate to myometrial contractility. In the past few decades, scientists have studied the mechanism of myometrial contractile activity using ultrasonographic and 3-dimensional reconstruction software [37–39]. The primary direction of the uterine contractile waves following ovulation is from the cervix toward the fundus [40]. This movement gradually decreases during the luteal phase, then reaches a nearly quiescent state by day 7 after HCG administration [37]. As we supposed, D5-ET shortens the “wandering” phase of the embryo prior to implantation, which decreases the likelihood of retrograde travel into the Fallopian tube. In addition, the blastocyst-stage embryo has a larger diameter than the previous stage, which probably allows it to be more resistant to the contractile waves of the uterine muscle.

The higher quality of the blastocyst may be another reason for the observed difference in EP rate. In nature cycles, aneuploidy could cause delayed migration, abnormal trophoblasts are more active and therefore implant at an earlier stage [41, 42].While in IVF-ET, Sekhon reported that there was a 60% decrease in the risk of ectopic pregnancy after IVF-PGS[43]. It has been reported that embryos with aneuploidy fail to develop in extended culture to the blastocyst stage [44, 45].Considering the decreased developmental potential of aneuploidy embryos, prolonging in vitro culture to day 5 allows the selection of chromosomally competent embryos [46, 47],and therefore leads to a reduced EP rate.

In the present meta-analysis, the impact of frozen-embryo use might be the most confounding factor. It is well known that estrogen promotes peristalsis in the wall of the Fallopian tube. It has also been shown that EP tends to occur when an excessive estrogen level or a much higher estrogen-to-progesterone ratio is present [48, 49]. Higher estrogen levels may promote tubal implantation through a deleterious impact on Fallopian-tube function [50]; the negative impact can be seen in ciliary-beat frequency [51], tubal-protein secretion [52], embryonic motility [53], and in the implantation process [54]. In our subgroup analysis, a reduced EP rate was seen for D5-ET in both the Fre-ET and Fro-ET populations. This information strengthened our hypothesis.

EP is a life-threatening complication of ART. Therefore, it is important to identify the related risk factors so that they may be avoided or decreased during the ART process. The present meta-analysis suggests strong support for D5-ET in IVF and ICSI. To the best of our knowledge, this is the first large-scale meta-analysis focused on the differing risk for EP between D3-ET and D5-ET. However, our study does have limitations. The majority of the studies included in this meta-analysis were retrospective; the potential for selection bias cannot be avoided, and the robustness of the findings is therefore limited. To correct for this potential bias, we separated out the RCTs and analyzed them independently. We observed a general trend toward a better outcome for D5-ET, but no statistical significance was proven. However, it should be noted that there were only 7 RCTs available for review, each with an insufficient sample size (theoretically, a total of 1920 subjects in each group are needed for 80% power, but no more than 374 subjects, each group, were included in the available RCTs). Therefore, the significance shown in retrospective studies and in our general results cannot be ignored. The benefits of D5-ET for lowering the EP risk should be fully considered in the ART process. A multicenter RCT using a large sample size is anticipated in the future. Besides, the studies involved in our meta-analysis stepped over a long time (since 1998 to 2015), and there might be some bias on embryo quality during culture/embryo transfer methodology during this long period, however, whether these bias affect our results can not be sure.

## Conclusion

The present study suggested that D5-ET can reduce the EP rate compared with D3-ET in IVF/ICSI cycles, no matter fresh or frozen-thawed embryo transfer was performed. D5-ET may be a better choice for decreased EP risk in ART treatment.

## Supporting Information

S1 File PRISMA 2009 Checklist. (DOC)

S1 Fig Risk of bias using Cochrane risk assessment tool for RCT. **A.** Summary of risk bias for each trial. Plus sign, low risk of bias; minus sign, high risk of bias; question mark, unclear risk of bias **B.** Risk of bias graph about each risk of bias item presented as percentages across all included studies.
(TIF)

S2 Fig Sensitivity analysis of omitting single study in turn. (TIF)

S3 Fig Publication bias of included study. In the current meta-analysis of ectopic pregnancy between day 3 and day 5 embryo transfer, the publication biases were evaluated by the Begg’s funnel plots and Egger’s linear regression. **A** Begg’s funnel plots of publication bias of EP rate. **B** Egger’s linear regression test of publication bias of EP rate, P = 0.10.
(TIF)

S1 Table Characteristics of women with clinical pregnancy in Center for Reproductive Medicine, Shandong University, 2010–2015. (DOCX)

S2 Table Newcastle–Ottawa quality assessment scale of the included retrospective cohort studies. (DOCX)

S3 Table Newcastle–Ottawa quality assessment scale of the included retrospective cohort studies. (DOCX)
