# Supplementary figures and images for: Reduced Ectopic Pregnancy Rate on Day 5 Embryo Transfer Compared with Day 3: A Meta-Analysis
Source: PLoS One. 2017 Jan 25;12(1):e0169837. doi: 10.1371/journal.pone.0169837 (PMC5266274; doi:10.1371/journal.pone.0169837)

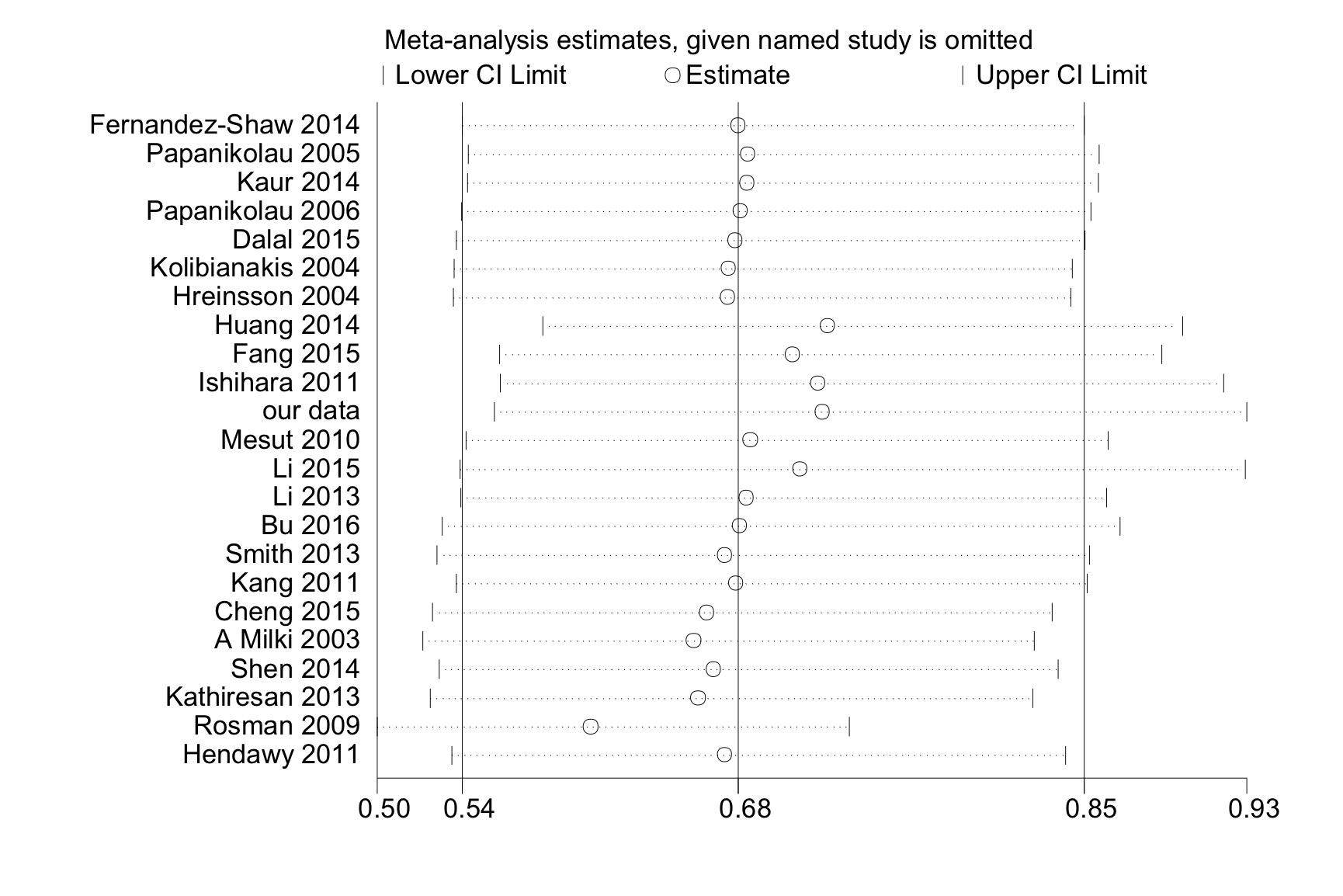

Supplement: S2 Fig — (TIF) [file pone.0169837.s003.tif]

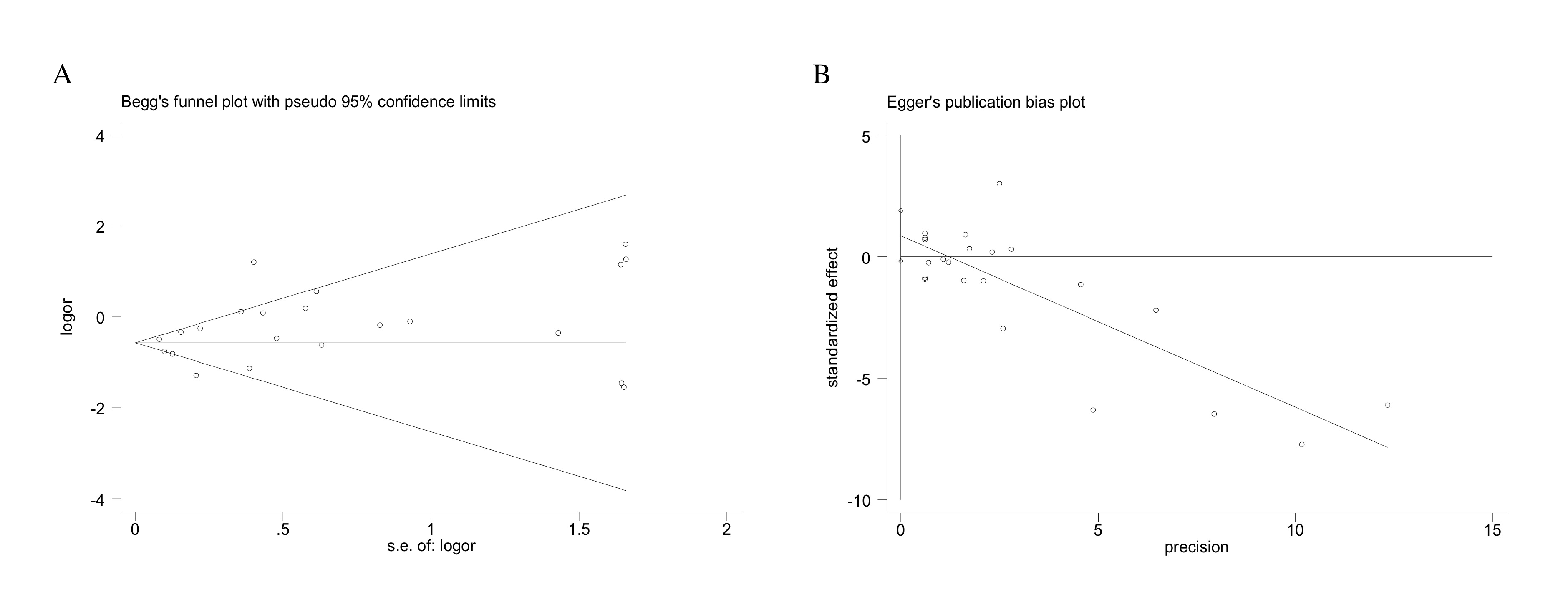

Supplement: S3 Fig — In the current meta-analysis of ectopic pregnancy between day 3 and day 5 embryo transfer, the publication biases were evaluated by the Begg’s funnel plots and Egger’s linear regression. A Begg’s funnel plots of publication bias of EP rate. B Egger’s linear regression test of publication bias of EP rate, P = 0.10. (TIF) [file pone.0169837.s004.tif]
