# Supplementary material for: Reduced Ectopic Pregnancy Rate on Day 5 Embryo Transfer Compared with Day 3: A Meta-Analysis
Source: PLoS One. 2017 Jan 25;12(1):e0169837. doi: 10.1371/journal.pone.0169837 (PMC5266274; doi:10.1371/journal.pone.0169837)
Supplement: S1 Table — (DOCX) [file pone.0169837.s005.docx]

**S1 Table** Characteristics of women with clinical pregnancyin Center for Reproductive Medicine ,Shandong University,2010-2015

|  | | Day 5 | Day 3 | P | P_adjusted_ |
| --- | --- | --- | --- | --- | --- |
| N | | 10768 | 20347 |  |  |
| EP rate[%(N)] | | 1.22 (131) | 2.57 (523) | <0.01* | <0.01* |
|  | EP in Fre-ET[%(N)] | 1.69 (24/1420) | 2.59（499/19265） | 0.04* |  |
|  | EP in Fro-ET[%(N)] | 1.1（107/9348） | 2.22（24/1082） | <0.01* |  |
| Age(yrs) | | 30.37±4.25 | 30.49±4.37 | 0.01* | 0.67 |
| BMI (kg/m^2^) | | 23.24±5.45 | 23.09±3.44 | <0.01* | 0.08 |
| Risk Factors of EP | |  |  |  |  |
|  | Previous pregnancy[%(N)] | 42.21（4545） | 42.96（8741） | 0.20 |  |
|  | Numbers of embryo transfer | 1.36±0.48 | 2.05±0.32 | <0.01* | 0.82 |
|  | Tubal disorders[%(N)] | 51.72（5569） | 55.31（11254） | <0.01* | <0.01* |
|  | Endometrial polyps[%(N)] | 8.89 （957） | 10.43（2123） | <0.01* | 0.42 |
|  | Intrauterine adhesions[%(N)] | 0.61 （66） | 0.48 （97） | 0.11 |  |
|  | Ovulatory dysfunction[%(N)] | 15.68（1688） | 11.45（2330） | <0.01* | 0.55 |
|  | Endometriosis[%(N)] | 2.60 （280） | 3.09 （628） | 0.02* | 0.99 |
|  | Adenomyosis[%(N)] | 0.19 （20） | 0.21 （43） | 0.63 |  |
|  | Uterine surgery history[%(N)] | 11.70（1260） | 12.99（2643） | <0.01* | 0.57 |
|  | Pelvic tuberculosis[%(N)] | 0.16 （17） | 0.14 （28） | 0.66 |  |
|  | Male factor[%(N)] | 73.03（3987） | 73.46（14946） | <0.01* | 0.03* |

Data was presented as as mean ± standard deviation or proportion;

The students’t test and chi-square test were applied to obtain group comparisons from continuous variables and categorical variable respectively; P-adjusted is the result of the binary logistic regression analysis .* P <0.05 was set as statistical significance;

EP, ectopic pregnancy; BMI, Body Mass Index ;Fre-ET, fresh embryo transfer ;Fro-ET, frozen-thawed embryo transfer
