# Supplementary material for: Reduced Ectopic Pregnancy Rate on Day 5 Embryo Transfer Compared with Day 3: A Meta-Analysis
Source: PLoS One. 2017 Jan 25;12(1):e0169837. doi: 10.1371/journal.pone.0169837 (PMC5266274; doi:10.1371/journal.pone.0169837)
Supplement: S2 Table — (DOCX) [file pone.0169837.s006.docx]

**S2 Table**  Newcastle–Ottawa quality assessment scale of the included retrospective cohort studies.

| Reference | Selection of exposed  group | Selection of non-exposed  group | Ascertainment  of exposure | Outcome  negative  at start | Comparability  by design and analysis | Outcome  assessment | Duration  of follow-up | Completeness  of follow-up | Score |
| --- | --- | --- | --- | --- | --- | --- | --- | --- | --- |
| Bu,2016 | * | * | * | * | ** | * | * | * | 9 |
| Cheng,  2015 | * | * | * | * | ** | * | * | * | 9 |
| Huang,  2014 | * | * | * | * | * | * | * | * | 8 |
| Smith,2013 | * | * | * | * | ** | * | * | * | 9 |
| Our data | * | * | * | * | * | * | * | * | 8 |
| A Milki ,  2003 | * | * | * | * | * | * | * | * | 8 |
| Ishihara,  2011 | * | * | * | * | * | * | * | * | 8 |
| Kang,2011 | * | * | * | * | * | * | * | * | 8 |
| Li,2015 | * | * | * | * | ** | * | * | * | 9 |
| Mesut, 2010 | * | * | * | * | * | * | * | * | 8 |
| Li,2013 | * | * | * | * | ** | * | * | * | 9 |
| Rosman,  2009 | * | * | * | * | * | * | * | * | 8 |

* Indicates that the feature is present; x, that the feature is absent. But for comparability by design this checklist awards maximum of two stars (**) ,one(*)or none of the feature is completely absent
