# Supplementary material for: Reduced Ectopic Pregnancy Rate on Day 5 Embryo Transfer Compared with Day 3: A Meta-Analysis
Source: PLoS One. 2017 Jan 25;12(1):e0169837. doi: 10.1371/journal.pone.0169837 (PMC5266274; doi:10.1371/journal.pone.0169837)
Supplement: S3 Table — (DOCX) [file pone.0169837.s007.docx]

**S3 Table** Newcastle–Ottawa quality assessment scale of the included retrospective case-control studies

| Reference | Case  representative | Control  representative | Selection of control | Ascertainment  of control | Comparability  by design and analysis | Ascertainment  of exposure | Method between case and control | Non response rate | Score |
| --- | --- | --- | --- | --- | --- | --- | --- | --- | --- |
| Fang,2015 | * | * | * | * | ** | * | * | * | 9 |
| Hendawy,2011 | * | * | * | * | * | * | * | * | 8 |
| Kathiresan,2013 | * | * | * | * | * | * | * | * | 8 |
| Shen,2014 | * | * | * | * | * | * | * | * | 8 |

* Indicates that the feature is present; x, that the feature is absent. But for comparability by design this checklist awards maximum of two stars (**) ,one(*)or none of the feature is completely absent
